# Approaches for identifying and measuring heteroresistance in azole-susceptible *Candida* isolates

**DOI:** 10.1128/spectrum.04041-23

**Published:** 2024-03-14

**Authors:** Cécile Gautier, Eli I. Maciel, Iuliana V. Ene

**Affiliations:** 1 Fungal Heterogeneity Group, Institut Pasteur, Université Paris Cité, Paris, France; University of Iowa, Iowa City, USA

**Keywords:** mycology, *Candida albicans*, drug responses, azoles, heteroresistance

## Abstract

Heteroresistance to antifungal agents poses a significant challenge in the treatment of fungal infections. Currently, the absence of established methods for detecting and measuring heteroresistance impedes progress in understanding this phenomenon in fungal pathogens. In response to this gap, we present a comprehensive set of new and optimized methods designed to detect and quantify azole heteroresistance in *Candida albicans*. Here, we define two primary assays for measuring heteroresistance: population analysis profiling, based on growth on solid medium, and single-cell assays, based on growth in liquid culture. We observe good correlations between the measurements obtained with liquid and solid assays, validating their utility for studying azole heteroresistance. We also highlight that disk diffusion assays could serve as an additional tool for the rapid detection of heteroresistance. These methods collectively provide a versatile toolkit for researchers seeking to assess heteroresistance in *C. albicans*. They also serve as a critical step forward in the characterization of antifungal heteroresistance, providing a framework for investigating this phenomenon in diverse fungal species and in the context of other antifungal agents. Ultimately, these advancements will enhance our ability to effectively measure antifungal drug responses and combat fungal infections.

**IMPORTANCE:**

Heteroresistance involves varying antimicrobial susceptibility within a clonal population. This phenomenon allows the survival of rare resistant subpopulations during drug treatment, significantly complicating the effective management of infections. However, the absence of established detection methods hampers progress in understanding this phenomenon in human fungal pathogens. We propose a comprehensive toolkit to address this gap in the yeast *Candida albicans*, encompassing population analysis profiling, single-cell assays, and disk diffusion assays. By providing robust and correlated measurements through both solid and liquid assays, this work will provide a framework for broader applications across clinically relevant *Candida* species. These methods will enhance our ability to understand this phenomenon and the failure of antifungal therapy.

## INTRODUCTION

Fungal infections remain inadequately addressed within the spectrum of human infectious diseases. A recent global survey reported a staggering annual occurrence of over 6.5 million cases of invasive fungal infections, contributing to more than 3.8 million fatalities (1). This burden significantly impacts human well-being, particularly immunocompromised individuals and those undergoing intensive medical care (2). Up to 70% of fungal infections are due to *Candida* species, posing a significant challenge due to their remarkable ability to evade antifungal treatment and host defenses (3, 4). *Candida* species are frequent causative agents for nosocomial infections and *Candida albicans* remains the most prevalent among these (5). Currently, there are no vaccines against fungal infections and only limited antifungal drugs are available for treatment (6, 7).

Antifungal resistance has been observed across all classes of drugs (4, 8), and it is associated with persistent infections and high mortality rates (9–11). The frequency of multidrug-resistant species is also increasing, with multiple reports of *Candida auris* and *Candida parapsilosis* causing outbreaks in medical facilities (11–14). Among antifungals, azoles are commonly used for the treatment of *Candida* infections (15, 16). This is owing to their accessibility, low toxicity, and broad spectrum of action. In *Candida* species, azoles function as fungistatic agents by inhibiting the activity of the enzyme Erg11 in the ergosterol biosynthesis pathway (17). Inhibition of this synthesis step leads to the accumulation of toxic intermediate sterols, resulting in the loss of cell membrane integrity and growth arrest (4). Azole resistance is often associated with increased expression of drug efflux pumps, mutations in genes regulating their expression, mutations in the drug target, or with increased copies of genes associated with drug resistance (4, 18).

In addition to *bona fide* drug resistance, population heterogeneity can serve as a bet-hedging strategy to increase the chances of survival upon drug exposure (19). Tolerance and heteroresistance have emerged as alternative mechanisms by which fungal pathogens can escape the consequences of drug exposure (20–27). Tolerance reflects the ability of an otherwise drug-susceptible subpopulation to grow at drug concentrations that are inhibitory for the rest of the population (28). It can be detected using disk diffusion assays or broth microdilution/minimum inhibitory concentration (MIC) assays, and it is measured as the residual fungal growth above drug inhibitory levels following extended incubation times (28). Tolerance has been linked to the inability of antifungals to clear fungal infections and is correlated with increased mortality (28, 29).

In contrast, heteroresistance implies the existence of a drug-resistant subpopulation within a drug-susceptible majority population. Such resistant subpopulations can grow efficiently in the presence of elevated drug concentrations but might lose this ability when the drug pressure is removed (30, 31). Heterogeneous antibiotic resistance was discovered in 1947 for *Haemophilus influenzae* (32). However, this phenomenon was first documented in 1970 in *Staphylococcus aureus,* where bacterial strains were tested in population analysis profiling (PAP) assays (33). Since then, it has been observed in multiple bacterial species with different antibiotics, in both bactericidal and bacteriostatic contexts (30, 34). Heteroresistance has been associated with the presence of increased copies of genes that confer resistance (31). The presence of such resistant bacterial subpopulations has been linked to an increased risk of recurrent infections (30, 34).

Recent work has described heteroresistance in fungal pathogens, notably in *Cryptococcus neoformans* (21–23, 27, 35), *Nakaseomyces glabrata* (formerly *Candida glabrata*) (20), and *Candida parapsilosis* (25) upon exposure to either azoles or echinocandins. Heteroresistance is likely to occur in other fungal species as well, and it could play critical roles in shaping antifungal treatment efficacy. *C. neoformans* heteroresistant cells were detected in the cerebrospinal fluid of fluconazole-treated cryptococcal meningitis patients, and their detection was associated with relapse from azole monotherapy (21, 23). *C. neoformans* azole heteroresistance was linked to the presence of an additional copy of chromosome 1, which harbors both *ERG11* and *AFR1*, genes encoding the azole drug target and a major drug efflux pump, respectively (22, 23). The higher expression of these genes is thought to contribute to the increased drug resistance, while increased efflux pump activity was positively associated with heteroresistance (23). Similarly, heteroresistance was associated with the failure of azoles to clear infection by *N. glabrata* (20). In this species as well, heteroresistant isolates displayed higher levels of drug efflux (20). Echinocandin-heteroresistant *C. parapsilosis* isolates were detected from breakthrough bloodstream infections in allogeneic hematopoietic cell transplant patients on micafungin prophylaxis (25). A machine learning model based on genomic features of heteroresistant isolates detected three single nucleotide variants ( SNVs) and one copy number variation (CNV) as potential predictors of echinocandin heteroresistance, but these were not further investigated (25). Overall, the significance of heteroresistance is becoming increasingly recognized for its impact on therapeutic outcomes (19, 30, 31), making this phenomenon an important parameter to consider when studying antifungal drug responses.

Heteroresistance is not currently measured in standard susceptibility assays as most approaches average whole population phenotypes. Detection methods have been developed to identify and characterize heteroresistance in bacterial populations, allowing for a more precise understanding of this phenomenon (30). The most established method is the PAP assay, although disk diffusion assay, E-tests, MIC, flow cytometry, time-kill assays, or combinations of these have also been used (30). Definitions of heteroresistance vary widely depending on the microbial species and the antibiotic. Frequently, resistance among heteroresistant subpopulations exhibits levels that are two to eight times greater than the resistance observed in the original population (30, 34). However, there is no clear consensus regarding the frequency and extent of resistance necessary for classifying a subpopulation as heteroresistant.

While we benefit from standardized susceptibility testing methods for resistance (in both clinical and research settings) and tolerance (in research labs) (26, 28, 36–38), antifungal heteroresistance is poorly characterized, and methods to quantify this phenomenon have not been formally established. Here, we define several methods to identify and quantify the existence of resistant subpopulations in *C. albicans* azole-susceptible isolates. We show that single-cell assays in liquid culture and PAP assays on solid medium can both detect and measure this phenomenon while demonstrating a strong correlation. We also argue for the use of short PAP assays and disk diffusion assays as rapid tools for quantifying or detecting heteroresistance, respectively. Additionally, we show that PAP assays are amenable to the testing of heteroresistance across a diverse set of yeast species. Together, these methods comprise a versatile toolkit for evaluating heteroresistance in *Candida* species.

## MATERIALS AND METHODS

### *C. albicans* strains and growth

All *C. albicans* isolates are listed in Table 1. *C. albicans* cells were cultured in liquid YPD medium [2% bacto-peptone, 1% yeast extract, and 2% dextrose (filter-sterilized)] overnight at 30°C with continuous shaking (200 rpm), unless otherwise specified. The number of cells in each culture was determined by measuring the optical densities of culture dilutions (OD_600_ nm) in sterile water using a Biotek Epoch 2 microplate reader (Agilent Technologies). The cultures were then diluted to the desired concentrations in sterile water.

**TABLE 1:** *C. albicans* and other species isolates used in this study^*a*^.

Name	Species	Susceptibility (FLC MIC_50_, µg/mL)	Average rate of heteroresistance (%)	Site of isolation	Reference/Source
*C. albicans* isolates					
CAY8847	*C. albicans*	4	<0.04	Oral cavity	(39)
CAY8856	*C. albicans*	2.67	<0.04	Oral cavity	(39)
CAY8762	*C. albicans*	>128		Oral cavity	(39)
P60002	*C. albicans*	>128		Bloodstream	(40)
SC5314	*C. albicans*	0.83	0.39	Bloodstream	(41)
CAY6420	*C. albicans*	0.67	0.30	Bloodstream	(28)
CAY6440	*C. albicans*	1.17	1.10	Bloodstream	(28)
CAY8836	*C. albicans*	0.67	2.71	Oral cavity	(39)
CAY8851	*C. albicans*	0.67	0.50	Oral cavity	(39)
CEC3544	*C. albicans*	0.83	0.50	Commensal	(42)
CEC4032	*C. albicans*	0.42	0.58	Superficial	(42)
CEC4499	*C. albicans*	0.67	3.97	Food spoilage	(42)
Other species					
4-7873A	*C. dubliniensis*	3.20	<0.04	Not available	(43)
4-7873C	*C. dubliniensis*	48	59.82	Not available	(43)
SA100	*C. dubliniensis*	0.79	39.39	Not available	(43)
DSY1448	*C. dubliniensis*	0.25	89.70	Oral infection	Dominique Sanglard
DSY1590	*C. tropicalis*	2	49.56	Oral infection	Dominique Sanglard
DSY2168	*C. tropicalis*	32	54.70	Oral infection	Dominique Sanglard
CP289	*C. orthopsilosis*	2	<0.04	Not available	(43)
CP331	*C. orthopsilosis*	0.60	0.07	Not available	(43)
R018-G3	*C. parapsilosis*	0.60	<0.04	Not available	(43)
R029-O4	*C. parapsilosis*	0.60	<0.04	Not available	(43)
ATCC96143	*C. metapsilosis*	3.60	<0.04	Not available	(43)
CP43	*C. metapsilosis*	2.80	<0.04	Not available	(43)
CA01	*C. auris*	6.67	<0.04	Auditory canal	(43)
CA02	*C. auris*	5.33	65.31	Burn wound	(43)
CA07	*C. auris*	5.33	37.44	Bloodstream	(43)
CA23	*C. auris*	41.60	9.49	Ear discharge	(43)
CA13	*C. haemulonii*	10.40	<0.04	Not available	(43)
CA15	*C. haemulonii*	2.67	0.09	Fish	(43)
CA11	*C. duobushaemulonii*	5.33	0.05	Bloodstream	(43)
CA12	*C. duobushaemulonii*	30.40	<0.04	Foot ulcer	(43)
CA14	*C. duobushaemulonii*	7.20	<0.04	Not available	(43)
CA16	*K. ohmeri*	8	<0.04	Not available	(43)
CA18	*C. lusitaniae*	1.33	<0.04	Not available	(43)
DSY2242	*N. glabrata*	32	0.26	Oral infection	(44)
DSY2243	*N. glabrata*	32	1.24	Oral infection	(44)
DSY552	*N. glabrata*	32	0.05	Oral infection	Dominique Sanglard
DSY1362	*N. glabrata*	1.67	1.75	Oral infection	Dominique Sanglard
CA19	*S.cerevisiae*	6.67	<0.04	Not available	(43)
CA20	*S. cerevisiae*	57.60	<0.04	Not available	(43)
BY4741	*S. cerevisiae*	22.86	<0.04	Not available	(43)

^*a*^ The rate of heteroresistance was calculated by taking the average growth on PAP assays at 48 h for fluconazole (FLC) concentrations equal to or greater than 10-fold the MIC_50_ of the parent isolate.

### MIC testing

These assays were performed as previously described (43). Briefly, ~2 × 10^5^ cells were cultured at 30°C with shaking (200 rpm) for 48 h in 96-well plates in a total volume of 125 µL of YPD with the following fluconazole (FLC) concentrations: 0, 0.125–128 μg/mL in two-fold increments. Cell densities were measured at 24 and 48 h using a BioTek Epoch 2 microplate reader. Susceptibility (MIC_50_) and tolerance (supra-MIC growth, SMG) values were determined after 24 and 48 h of growth, respectively, as previously described (36, 43). Briefly, MIC assays consist of determining the minimum inhibitory drug concentration at 50% growth inhibition relative to untreated control wells (MIC_50_). SMG is measured by averaging the amount of growth in wells above MIC_50_ values, normalized to the growth in wells without drug. All assays were performed with three biological replicates.

For MIC assays performed in RPMI, we followed the Clinical and Laboratory Standards Institute (CLSI) guidelines (45). Briefly, a similar protocol to the one described above was followed with the following changes: cells were grown in RPMI medium (1% RPMI with glutamine, 3% MOPS, and 2% dextrose, pH 7) at 35°C without shaking for the duration of the assay (48 h).

### Disk diffusion assays

10^5^ *Candida* cells were evenly spread on YPD agar [2% bacto-peptone, 1% yeast extract, 2% dextrose (filter-sterilized), and 2% agar] or RPMI agar (1% RPMI with glutamine, 3% MOPS, 2% dextrose, and 2% agar, pH 7) plates using glass beads. A disk containing 25 µg fluconazole [Liofilchem (9166) or Oxoid (CT1806B)] was positioned at the center of the agar plate. The plates were incubated at 30°C for 48 h, and images were captured using a PhenoBooth+ instrument (Singer). Susceptibility and tolerance were determined by measuring RAD_20_ (the radius of the zone of drug inhibition, measured at 20% FLC inhibition) and FoG_20_ (the fraction of growth within the area of drug inhibition, calculated using the 20% FLC inhibition cutoff), respectively, using the R package *diskImageR* (36). At least three independent disk diffusion assays were performed for each isolate; representative images are shown.

### PAP assays

YPD or RPMI agar was prepared with various FLC concentrations (0, 0.5, 1, 2, 4, 8, 16, 32, 64, and 128 µg/mL). Yeast cells were grown overnight in YPD medium and diluted to a concentration of 1 × 10^6^ cells/mL. Four cell dilutions were prepared: 10^5^, 10^4^, 10^3^, and 10^2^ cells/mL. Each YPD-FLC plate was then seeded with five spots of 5 µL from each cell dilution. The plates were incubated at 30°C and imaged at 24 and 48 h. The number of non-confluent colonies was tallied for each cell concentration to determine the fraction of cells able to grow at each FLC concentration. These numbers were then normalized to the number of colonies growing on YPD plates without FLC. The fraction of cells was plotted across the different FLC concentrations. All PAP assays were performed with at least three biological replicates. The rate of heteroresistance was calculated by taking the average growth on PAP at 48 h for FLC concentrations equal to or greater than 10-fold the MIC_50_ of the parent isolate. When such concentrations exceed the dynamic range of the assay, the maximum FLC concentration (128 µg/mL) was used instead.

The area under the curve (AUC) and the corresponding standard error were calculated using GraphPad Prism (v10). Only FLC concentrations equal to or greater than the MIC_50_ values were considered for AUC calculations. For both *C. albicans* and other species, the AUC is shown relative to the AUC of the *C. albicans* SC5314 reference isolate.

To assess the FLC susceptibility of *C. albicans* cells growing on PAP assays, five colonies were randomly selected from FLC 128 µg/mL plates for each isolate. Only three colonies could be recovered for isolate CAY8851. From these colonies, ~2 × 10^5^ cells were grown directly in 96-well plates at 30°C with shaking (200 rpm) for 24 h in a total volume of 125 µL of YPD with either no drug or with FLC. The FLC concentrations used were 8- and 16-fold higher than the parent MIC_50_. Cell densities were measured at 0 and 24 h using a BioTek Epoch 2 microplate reader and normalized to those measured in wells without FLC.

To assess the FLC susceptibility of cells from other species growing on PAP assays, 16 colonies were randomly selected from FLC 128 µg/mL plates. Only five colonies could be recovered for *Candida orthopsilosis* isolate CP331. From these colonies, ~2 × 10^5^ cells were grown directly in 96-well plates, and MIC assays were performed (as described above). For the validation of YPD PAP colonies in RPMI assays, a total of 15 colonies from three biological replicates per isolate were tested using the RPMI MIC method described above.

### Short PAP assays

*C. albicans* cell densities were adjusted to 10^6^ cells/mL, from which three subsequent dilutions were prepared: 10^5^, 10^4^, and 10^3^ cells/mL. Three droplets of 5 µL from these cell suspensions were plated onto YPD agar supplemented with 0 or 128 µg/mL FLC. The plates were incubated at 30°C and imaged at 24 and 48 h to determine the number of non-confluent colonies from the different cell dilutions. These numbers were normalized to the observed growth on plates without FLC, and the fraction of growth at 128 µg/mL FLC was plotted for each isolate. All assays were performed with three biological replicates.

### Single-cell assays

*C. albicans* cultures were diluted to 10^2^ cells/mL, and 96-well plates were seeded with 10 µL of cell suspension and 185 µL of YPD or YPD + FLC (at a concentration 10-fold higher than the MIC_50_ of the respective strain). A total of 384–768 wells were tested for each biological replicate, and single-cell assays were performed with three to five biological replicates. The number of cells inoculated per well was normalized by plating 12 aliquots of 10 µL from the inoculum for the determination of colony-forming units (CFUs). The CFU counts indicated that, across experiments, the wells were seeded with 0.83–1.167 cells per well (number termed here normalized inoculum). The normalized inoculum was used to determine the total number of cells seeded per biological replicate. The plates were incubated at 30°C with shaking (200 rpm) for a total of 96 h. At 48, 72, and 96 h, the plates were mixed by vortexing, and their optical density (OD_600_) was read using a Biotek Epoch 2 microplate reader. Wells with OD_600_ ≥ 0.5 were considered for subsequent analysis, and their frequency was determined relative to the total number of wells inoculated. Thus, to calculate the fraction of wells with proficient growth (OD_600_ ≥ 0.5), the following formula was used:


$$fraction\;of\;wells\;with\;proficient\;growth\;\left(\%\right)=\;\left(normalized\;inoculum\right)\times \left(\;\frac{number\;of\;wells\;with\;proficient\;growth}{number\;of\;wells\;inoculated}\;\times 100\right)$$

After 96 h, 8–16 wells with proficient growth were randomly selected for FLC susceptibility testing. Fifty- microliter aliquots from these wells were grown in 5 mL YPD overnight, and these cultures were used in MIC assays (as described above). Both susceptibility (MIC_50_) and tolerance (SMG) were determined. Next, the fraction of wells displaying MIC_50_ values 10-fold higher than the MIC_50_ of the parent strain, termed frequency of recovery, was determined relative to the number of wells tested. The rate of heteroresistance was calculated by multiplying the fraction of wells with proficient growth with the frequency of recovery.


rate of heteroresistance (%)=fraction of wells with proficient growth (%) ×frequency of recovery (%) ×100

A similar protocol was followed for single-cell assays in RPMI, with several modifications. First, due to the slower cell growth in RPMI medium, the incubation time was extended from 96 to 144 h. Additionally, as growth levels were overall lower than those observed in YPD, the growth threshold for wells with proficient growth was adjusted from an OD_600_ ≥ 0.5 to an OD_600_ ≥ 0.35. For the validation of cells in RPMI MIC assays, a total of 21 wells from two biological replicates were tested in susceptibility assays using the RPMI MIC method described above.

### Statistical analyses

Correlation analyses were performed using two-tailed nonparametric Spearman tests. *P* values < 0.05 were denoted as significant. AUC values and statistical analyses were obtained using GraphPad Prism (v10).

## RESULTS

### Disk diffusion assays can detect the presence of heteroresistant colonies

To detect the presence of potential heteroresistant cells in azole-susceptible *C. albicans* isolates, we selected 10 strains with relatively high susceptibility and 2 highly resistant strains as controls (P60002 and CAY8762). Most of the strains represent clinical isolates recovered from diverse infection sites, including bloodstream, oral, and superficial infections, and include a commensal and a food spoilage isolate (Table 1). This set of isolates was tested on both liquid MIC assays as well as disk diffusion assays to evaluate their FLC susceptibility and tolerance. On liquid MIC assays, susceptibility levels were determined following 24 h of growth by identifying the FLC concentration resulting in 50% growth inhibition (MIC_50_). In addition, prolonged growth (48 h) allows the determination of tolerance by measuring the SMG, or the supra-MIC growth, which reflects the amount of growth above MIC_50_ levels relative to the growth in wells without drug (28). Disk diffusion assays are another efficient way to assess drug susceptibility and tolerance, based on the radius of the area of drug inhibition (RAD_20_, a measure of drug susceptibility at 20% drug inhibition) and the fraction of cell growth in the zone of inhibition relative to the growth in areas not exposed to FLC (FoG_20_, calculated using the 20% FLC inhibition cutoff, see Materials and Methods), respectively. These assays confirmed the high resistance of isolates P60002 and CAY8762 and the high susceptibility of the 10 remaining isolates, whose MIC_50_ values ranged from 0.83 to 4 µg/mL (Fig. S1A and C), values that are below or at the clinical breakpoint for FLC resistance (37).

**Fig 1:**
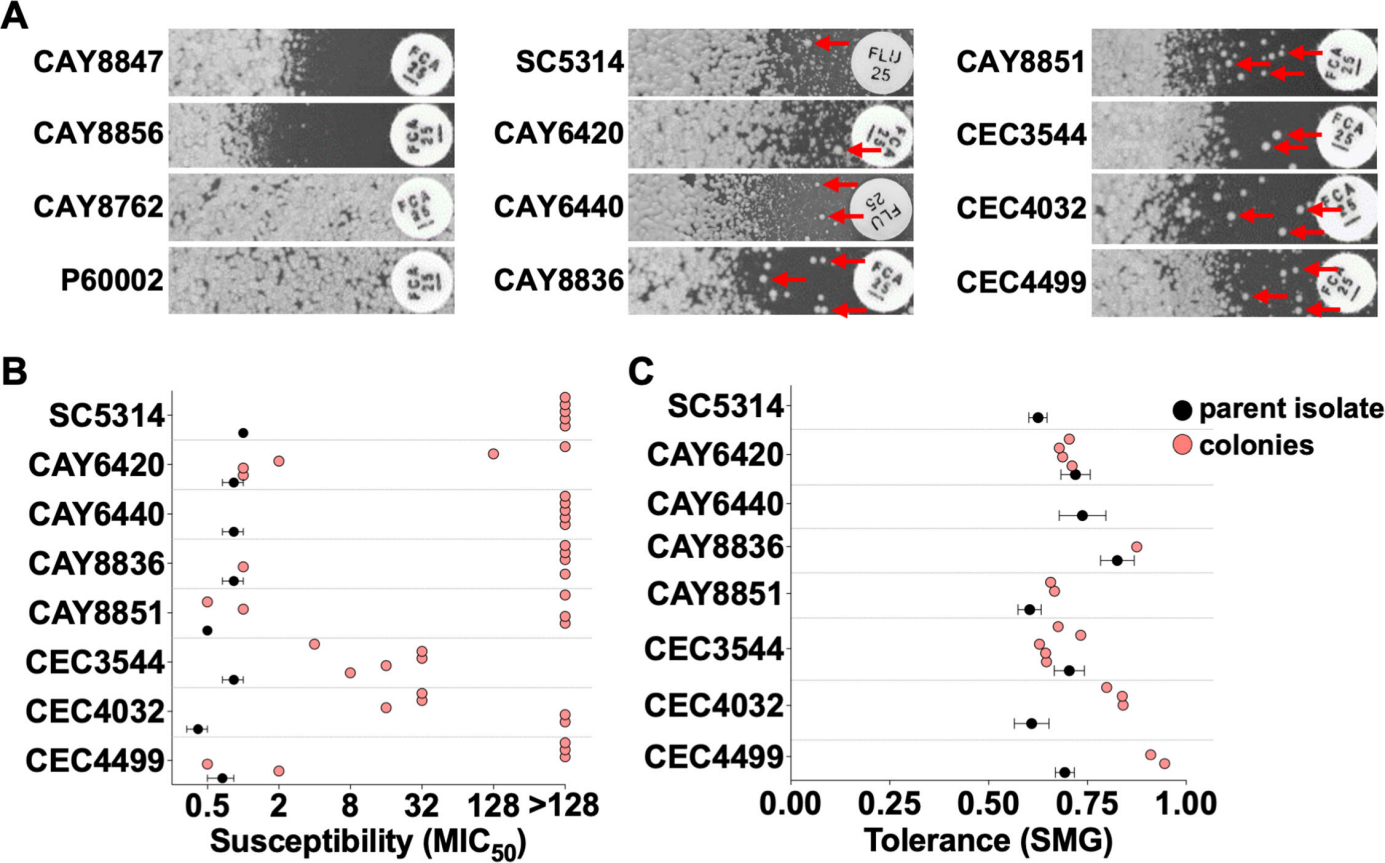
Disk diffusion assays can detect the presence of heteroresistant cells. (**A**) Images show cross-sections of disk diffusion assays of *C. albicans* isolates displaying different numbers of heteroresistant colonies in the zone of inhibition (FLC drug disk, 25 µg). Isolates CAY8847 and CAY8856 show a clear area of inhibition without any colonies present, while isolates CAY8762 and P60002 were fully resistant and grew to the edge of the FLC disk. Red arrows indicate potential heteroresistant colonies detected in the other eight isolates. FLC susceptibility (B, MIC_50_) and tolerance (C, SMG determined from MIC assays) levels of parent isolates (black) and five randomly selected large colonies (salmon) from each of the eight isolates. Error bars show the standard error of the mean (S.E.M.) of three biological replicates.

In addition, the 10 susceptible isolates displayed diverse tolerance levels on both liquid and solid assays, ranging from 0.27 to 0.83 for SMG (Fig. S1B and D), indicating tolerant subpopulations of variable sizes. Indeed, the zone of drug inhibition of disk diffusion assays is often populated with tolerant colonies of small size due to their slower growth in the presence of drug (28). These colonies were previously shown to have similar susceptibility and tolerance levels as the originating population (28). We were surprised to detect colonies of a larger size within this zone of inhibition for several of the strains examined, colonies that appeared uninhibited by the drug (marked with red arrows, Fig. 1A). Interestingly, assessing the susceptibility of these large colonies revealed that their resistance levels were 1.2- to 256-fold higher than those of the corresponding parent population (Fig. 1B), indicating the presence of heteroresistant subpopulations of cells. Inspired by bacterial definitions (30), we classified heteroresistant colonies as those that displayed at least 10-fold higher MIC_50_ levels relative to the MIC_50_ of the parent population. Using this classification, 30 out of 40 (75%) of the large colonies tested were consistent with a heteroresistant profile. CAY8847 and CAY8856 did not display any large colonies in the zone of drug inhibition (Fig. 1A), and therefore no colonies could be tested. We also determined the tolerance (SMG) levels of the large colonies with MIC_50_ levels lower than or equal to 128 µg/mL (the highest FLC concentration tested in MIC assays). Across lineages, the large colonies displayed similar (CAY6420, CAY8836, CAY8851, and CEC3544) or higher tolerance levels (CEC4032 and CEC4499) relative to the tolerance of the parent isolates (using SMG measurements at 48 h from MIC assays, Fig. 1C), indicating that increased tolerance alone could not explain the increased colony size of these colonies.

These results illustrate that disk diffusion assays represent a rapid method to detect the presence of heteroresistant colonies, but this method cannot accurately quantify this phenomenon. One major drawback is that high tolerance levels can yield a high number of tolerant colonies, which can mask the presence of heteroresistant colonies making these difficult to identify and isolate. Consequently, the precise quantification of heteroresistance is not possible using this method, highlighting the need for more robust and quantitative approaches.

### PAP assays can accurately measure *C. albicans* azole heteroresistance

**Fig 2:**
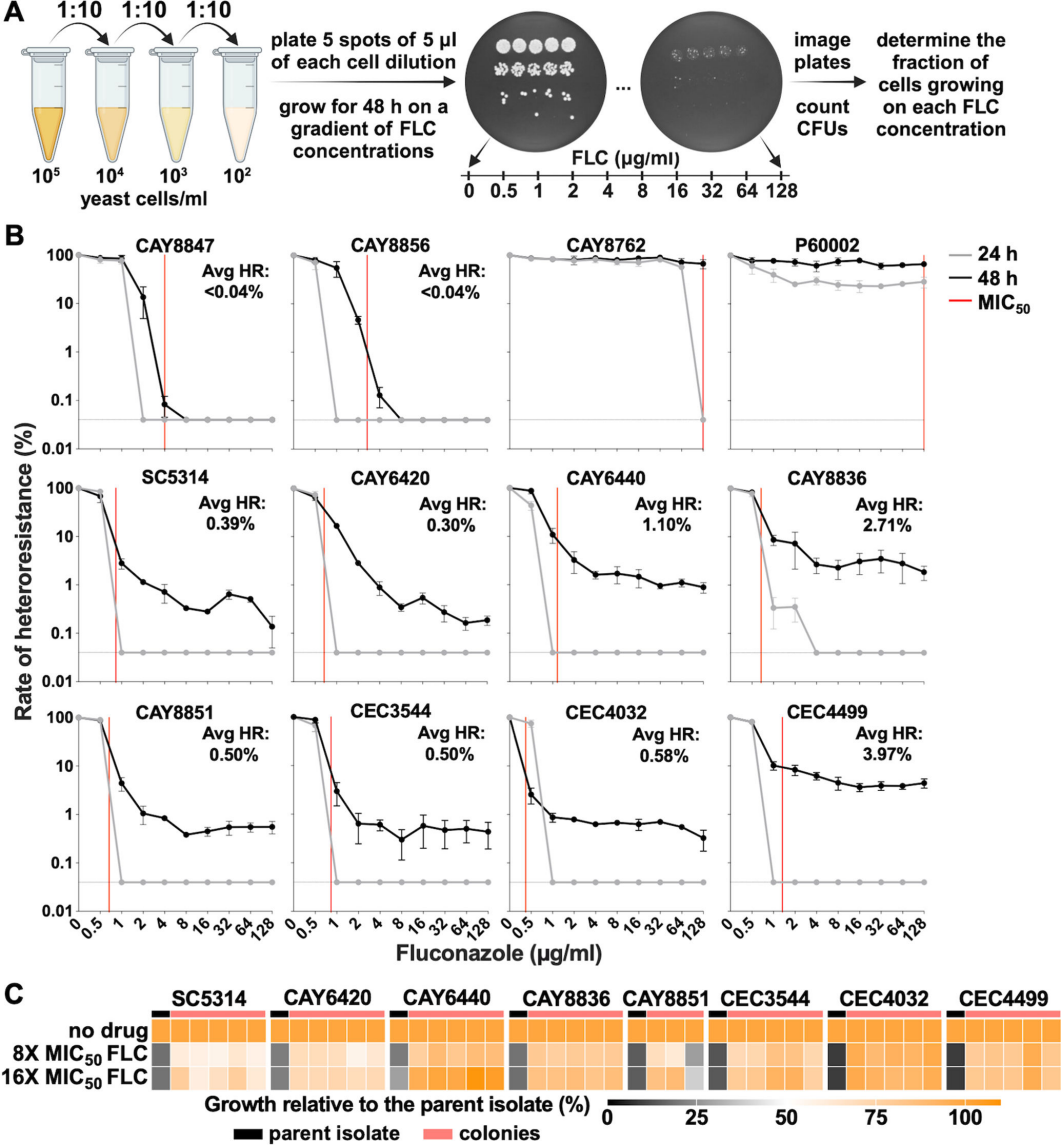
PAPs can accurately quantify azole heteroresistance. (**A**) *C. albicans* cultures were serially diluted and plated on YPD plates without antifungal or supplemented with a gradient of FLC concentrations (0.5–128 µg/mL in two-fold dilutions). The plates were incubated for 48 h and imaged at 24 and 48 h for CFU determination. The fraction of cells growing at each concentration was determined by counting the number of colonies growing on FLC relative to the number of colonies present on plates without FLC. (**B**) PAP profiles of the 12 strains examined in this study. Heteroresistance levels were calculated by taking the average growth on PAP at 48 h for FLC concentrations equal to or greater than 10-fold the MIC_50_ of the parent isolate (Avg HR). The dotted lines indicate the detection limit of the assay (0.04% of the population), and the red lines show MIC_50_ levels. Error bars show the standard error of the mean (S.E.M.) of three biological replicates. (**C**) Susceptibility measurements of up to five randomly selected colonies (salmon) recovered from FLC 128 µg/mL plates. Susceptibility levels of parent isolates (black) are included for reference. Susceptibility was determined by assessing cell growth at FLC concentrations 8- and 16-fold higher than the MIC_50_ of the corresponding parent isolate.

In bacterial populations, PAP assays are considered the gold standard for measuring both the size of heteroresistant subpopulations and the resistance level (30, 46). This resource-intensive method is not used in clinical laboratories. Instead, research laboratories employ it retrospectively to identify heteroresistance in cases of treatment failure. For fungal pathogens, this assay has been adapted for *N. glabrata* (20), *C. neoformans* (23), and *C. parapsilosis* (25) in the context of FLC and echinocandin treatment. PAP assays consist of quantifying the number of resistant colonies growing across a range of drug concentrations relative to those growing on plates without drug. We performed PAP assays for the 12 strains selected for this study using FLC concentrations of 0–128 µg/mL and imaged the plates for CFU quantification (Fig. 2A). The number of cells plated ranged from 0.5 to 500 cells per spot with a total of five spots per dilution (resulting in 2.5 to 2,500 cells plated for each dilution). PAP profiles were obtained by plotting the percentage of CFUs observed across each FLC concentration relative to the CFUs on plates without antifungal. Previous studies have used 1–5 days for incubation of these assays, as well as different temperatures [30°C or 35°C (20, 23, 25)]. To avoid colony filamentation, PAP plates were incubated at 30°C, and CFUs were quantified at both 24 and 48 h (Fig. 2A). After 24 h, no colony growth could be observed above liquid MIC_50_ levels, consistent with their FLC susceptibility profiles. CAY8847 and CAY8856 displayed growth inhibition at drug concentrations approximately two-fold lower than MIC_50_ concentrations (Fig. 2B), which could be due to the differences in susceptibility when measured in liquid versus solid assays. As expected, resistant isolates included as controls displayed proficient growth at both time points, although both required the full 48 h period for most of the population to grow at the highest FLC concentrations (Fig. 2B). CAY8847 and CAY8856 showed no additional growth above MIC_50_ levels, which was consistent with the absence of heteroresistant colonies in disk diffusion assays (Fig. 1A and 2B). In contrast, the remaining eight strains displayed subpopulations with robust growth at drug concentrations well exceeding MIC_50_ levels (Fig. 2B). These subpopulations varied in size between 0.3% and 4.08% (note that the detection limit of the PAP assay is 1/2,500 cells, equivalent to 0.04%). Surprisingly, strains displaying growth above MIC_50_ levels were characterized by a steady plateau-like growth of the heteroresistant subpopulation across FLC concentrations, indicating that this phenomenon is likely independent of drug concentration (Fig. 2B). Isolates displaying detectable growth at concentrations equal to or greater than 10 times MIC_50_ of the parent isolate were classified as heteroresistant, and the average size of the subpopulation growing across these concentrations was calculated as the rate of heteroresistance (see Materials and Methods). Calculating the AUC of PAP assays revealed that the AUC paralleled the average heteroresistance rate measurements; therefore, both parameters can be used to measure this phenomenon (Fig. S1E).

To validate the presence of resistant colonies among heteroresistant subpopulations, up to five colonies growing on 128 µg/mL FLC PAP plates were randomly selected and their FLC susceptibility was determined. To facilitate these experiments, we measured their growth in the presence of no drug, 8-fold, and 16-fold higher FLC concentrations relative to the MIC_50_ of the parent isolate. All colonies tested (37/38, 97%), except for one (originating from isolate CAY8851 and for which only three colonies could be recovered), were able to grow efficiently at FLC concentrations 16-fold higher than the MIC_50_ of parent isolate, indicating that they were at least 16-fold more resistant than corresponding parent strains and therefore could be classified as heteroresistant (Fig. 2C). We also tested whether the rate of heteroresistance was linked to other drug responses such as the susceptibility (MIC_50_) or the tolerance (SMG) of the parent isolate. However, no significant correlation was observed between the average heteroresistance rate and either of these parameters (Spearman correlation test, *r* = −0.585, *P* = 0.079 for MIC_50_; *r* = 0.596, *P* = 0.075 for SMG; Fig. S2A and B), indicating that these phenomena are likely independent of each other. Overall, these data establish that *C. albicans* azole heteroresistance can be accurately detected and quantified following 48 h of growth at 30°C by using YPD PAP assays.

**Fig 3:**
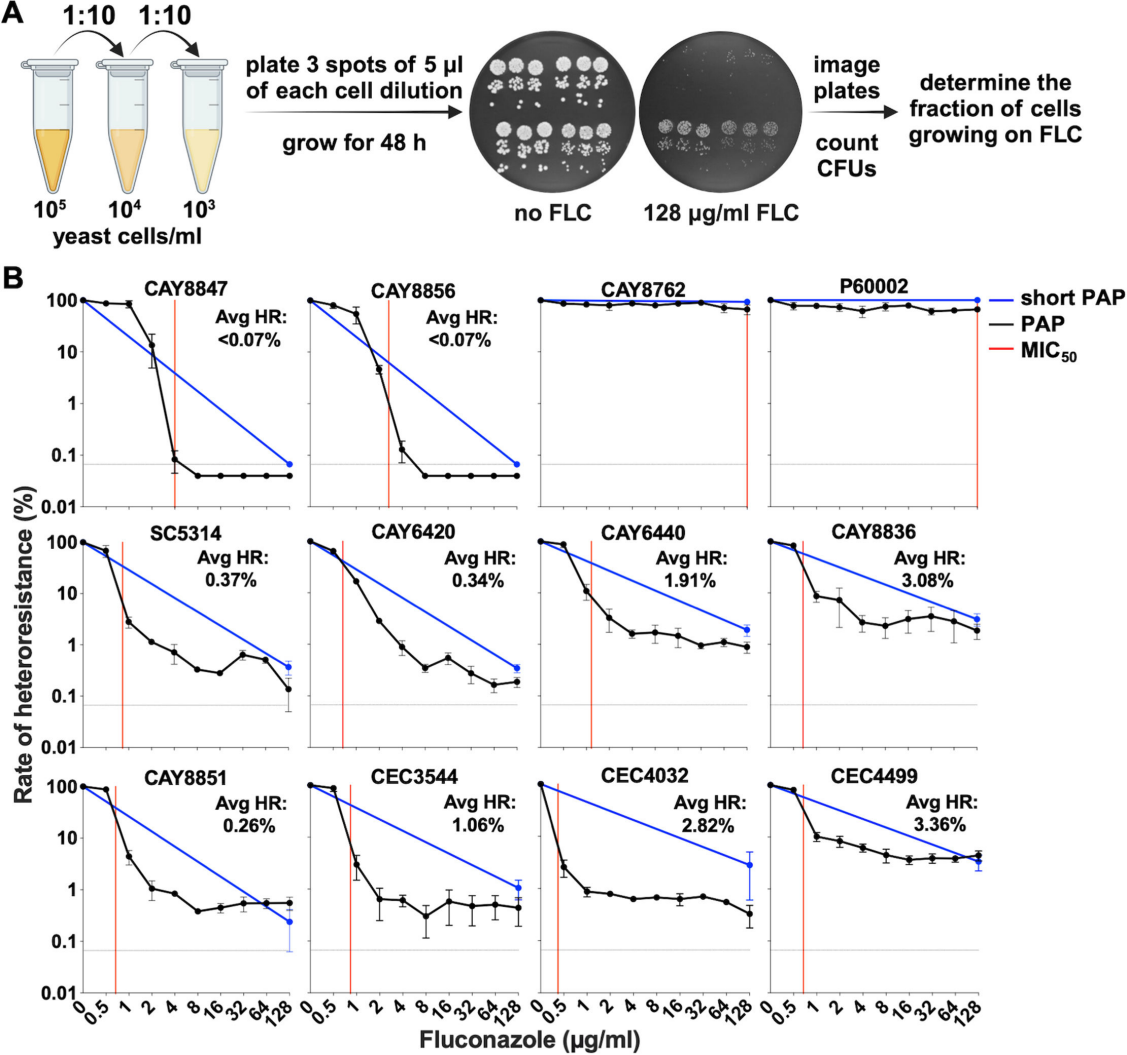
Short PAP assays represent an efficient method for measuring azole heteroresistance. (**A**) *C. albicans* cultures were serially diluted and plated on YPD plates without antifungal or supplemented with 128 µg/mL FLC. The plates were incubated for 48 h and imaged for CFU determination. The fraction of cells growing on FLC was determined relative to the growth on plates without drug. (**B**) Short PAP profiles of the 12 strains examined in this study. The dotted lines indicate the detection limit of the assay (0.067% of the population). Conventional PAP assays are included for reference. Avg HR, average heteroresistance rates measured with short PAP assays. Red lines show MIC_50_ levels, and error bars show the standard error of the mean (S.E.M.) of three biological replicates.

As PAP assays involve a large volume of work, we reasoned this method could not be employed for the screening of large numbers of fungal isolates. Given that heteroresistance levels appeared independent of drug concentration (Fig. 2B), we tested whether a single drug concentration would be sufficient to assess the size of the heteroresistant subpopulation. We selected the highest drug concentration employed in PAP assays and plated different cell dilutions on either no drug or 128 µg/mL FLC while maintaining a low detection limit (1/1,500 cells, equivalent to 0.067%). Decreasing both the number of cell dilutions and spots plated for each dilution allowed testing of up to four isolates per plate, therefore, reducing the volume of work by ~20-fold (while 10 plates are needed for one isolate using conventional PAP assays, only 2 plates are needed for four isolates using this approach, Fig. 3A).

The fraction of the heteroresistant cell population was determined for each of the 12 strains after 48 h of growth using this approach. No growth was observed for strains CAY8847 and CAY8856 (Fig. 3B), consistent with the results observed with disk diffusion and PAP assays. In contrast, resistant strains CAY8762 and P60002 displayed high levels of growth on 128 µg/mL FLC plates (Fig. 3B). For the eight heteroresistant isolates, the size of the subpopulation enumerated using the 128 µg/mL FLC plates varied between 0.34% and 3.36% (Fig. 3B), rates that were highly similar to those measured using conventional PAP assays. Indeed, a positive correlation was observed between the rates of heteroresistance determined with PAP and short PAP assays (Spearman correlation test, *r* = 0.717, *P* = 0.024; Fig. S2C). Thus, short PAP assays can be used for a faster assessment of *C. albicans* azole heteroresistance.

### Single-cell assays as a method for quantifying heteroresistance in liquid assays

**Fig 4:**
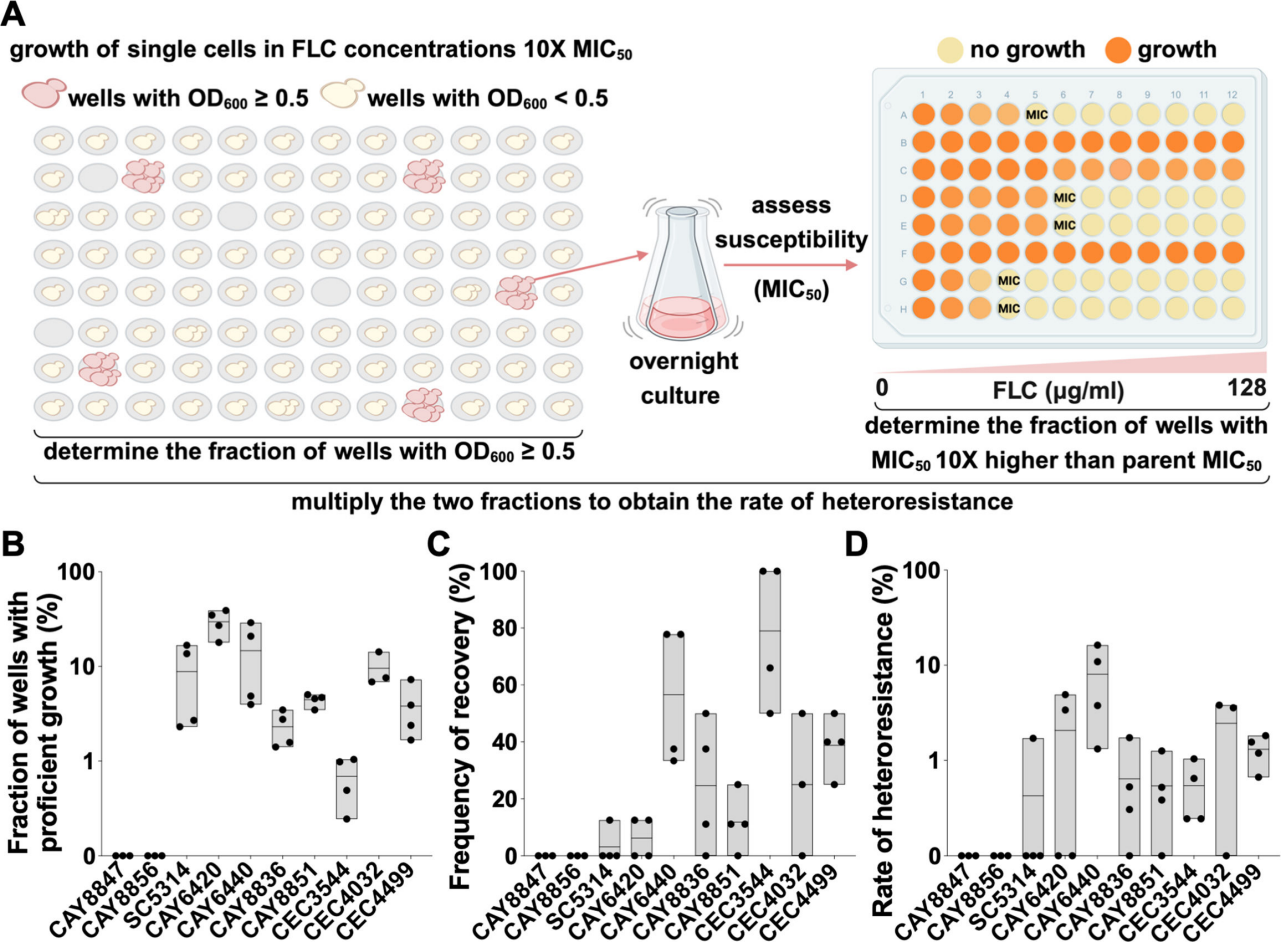
Single-cell assays can measure the frequency of heteroresistance in liquid culture. (**A**) Schematic of single-cell assays, whereby individual cells were grown in FLC concentrations 10-fold higher than the MIC_50_ of the parent isolate. A subset of cells recovered from wells showing robust growth were further tested in susceptibility assays. Heteroresistance rates were calculated by multiplying the fraction of wells with proficient growth with the rate of recovery of resistant isolates from the tested wells. Histograms show the fraction of wells with proficient growth (OD_600_ ≥ 0.5, **B**), the fraction of heteroresistant isolates recovered from the tested wells (**C**), and the rate of heteroresistance for each isolate (**D**). Experiments were performed with three to four biological replicates, each with at least 384 cells tested. Lines show average values.

We also developed a liquid assay to measure the frequency of heteroresistance within drug-susceptible populations. The assay involves growing hundreds of single cells in individual wells (384–768 wells per biological replicate, equivalent to a detection limit of 0.13%–0.26%) in the presence of a drug concentration 10-fold greater than the MIC_50_ of the parent isolate (Fig. 4A). Yeast cells were incubated for 96 h during which their growth was monitored at regular intervals. Wells with optical densities (OD_600_) equal to or greater than 0.5 after 96 h of growth were considered for later analyses, and their proportion was determined relative to the total number of cells assayed. The susceptibility of the cells from a subset of growing wells was then assayed to determine if they represent heteroresistant cells (Fig. 4A). The set of 10 susceptible strains was assayed using this approach, and it became apparent that they showed a diversity of growth profiles, from isolates CAY8847 and CAY8856 displaying no growth to isolates displaying a significant proportion of wells with proficient growth. For example, 29.81% of the CAY6420 wells showed robust growth by 96 h (Fig. 4B). In contrast, only 0.69% of the CEC3544 wells reached an OD_600_ ≥ 0.5 (Fig. 4B).

From the wells displaying proficient growth, 8–16 wells per biological replicate were randomly selected to test the FLC susceptibility of the cells growing in these wells. The exception was isolate CEC3544 for which fewer than eight wells were available and for which all wells were tested. Almost half of the isolates tested showed MIC_50_ levels greater than those of the parent isolate (≥1.25× fold change, 115/247 wells, 46.6%), with only a minority of isolates showing decreased resistance (≤0.75× fold change, 6/247 wells, 2.4%; Fig. S3A). For isolates where tolerance levels could be calculated, MIC assays revealed that a subset of isolates also had increased SMG levels relative to parent isolates (≥1.25×, 53/150 wells, 35.3%), while only one isolate showed the opposite trend (≤0.75×, 1/150 wells, 0.67%; Fig. S3B). The frequency of wells displaying FLC resistance levels equal to or greater than 10-fold the MIC_50_ of parent isolates was determined. Here, the rate of recovery of heteroresistant cells varied widely, from 3.13% as seen with SC5314 to 79% seen with CEC3544 (Fig. 4C).

Finally, the fraction of wells with proficient growth and the frequency of recovery of heteroresistant cells were multiplied to yield the rate of heteroresistance, resulting in diverse levels across the eight isolates, from 0.43% (for SC5314) to 8.07% (for CAY6440) (Fig. 4D). These rates showed a positive correlation to those measured with PAP assays (Spearman correlation test, *r* = 0.657, *P* = 0.045; Fig. S2D), indicating that this method can be reliably used to measure FLC heteroresistance. Given the diversity of susceptibility and tolerance levels observed with isolates recovered from single-cell assays, our data also indicate that this approach can reveal a substantial capacity for evolution under drug pressure relative to bulk assays where diverse lineages are likely to be masked by whole population phenotypes.

### RPMI solid medium does not accurately reflect different azole responses

In clinical settings, susceptibility testing is conventionally performed under CLSI conditions in RPMI medium (45). To determine whether heteroresistance could also be detected on RPMI, we selected three isolates representative of different categories: a susceptible isolate that does not form heteroresistant cells (CAY8847), a susceptible isolate with high heteroresistance rates on both PAP and single-cell assays (CAY6440), and a resistant isolate (P60002, see Fig. 1 to 4). We first tested the susceptibility of these isolates on RPMI liquid MIC assays (at 35°C, pH 7, without shaking), which revealed similar patterns in MIC_50_ and SMG levels to those observed in YPD (Fig. S4A and B). However, testing of these isolates on RPMI disk diffusion assays revealed minimum differences between strains, with the three isolates showing similar susceptibilities (RAD_20_) and tolerance levels (FoG_20_), which was in stark contrast with the results obtained on YPD (Fig. S4C through E). Surprisingly, none of the isolates displayed a clear area of inhibition nor displayed any heteroresistant colonies in this area (Fig. S4C), indicating that different azole responses cannot be discriminated on RPMI solid medium. We also performed single-cell assays for the heteroresistant isolate (CAY6440) using RPMI medium and incubation at 35°C. We observed growth levels similar to those observed in YPD (Fig. S4F), despite using a lower growth cutoff (OD_600_ ≥ 0.35 instead of 0.5) and an extended incubation time (144 instead of 92 h) due to the reduced growth rates observed on RPMI (Fig. S4F). However, the susceptibility of the cells growing in these wells could not be tested due to the very high level of filamentation observed.

Additionally, we sought to determine whether the susceptibility of heteroresistant cells growing on YPD PAP and YPD single-cell assays could be validated using CLSI testing methods. Therefore, we recovered CAY6440 colonies from PAP plates containing 128 µg/mL FLC and tested their FLC susceptibility in RPMI MIC assays. All colonies tested (15/15) displayed a >10-fold higher MIC_50_ relative to the parent strain, demonstrating that isolates recovered from this assay meet the resistance criteria observed by the CLSI method (Fig. S4G). Similarly, most isolates (18/21) obtained from YPD single-cell assays displayed a >10-fold higher FLC resistance in RPMI MIC assays (Fig. S4G), confirming that this assay can identify subpopulations with increased resistance levels. The tolerance (SMG) levels of the isolates examined from both assays were similar to those of parent strains (Fig. S4H). Taken together, these results indicate that heteroresistant isolates identified using these methods form subpopulations whose high resistance levels can be detected in MIC assays using both YPD and CLSI methods. Moreover, detection methods relying on RPMI agar cannot reliably distinguish between susceptibility, tolerance, and heteroresistance and therefore are not recommended for identifying these drug responses.

### Multiple *Candida* species display azole heteroresistance

**Fig 5:**
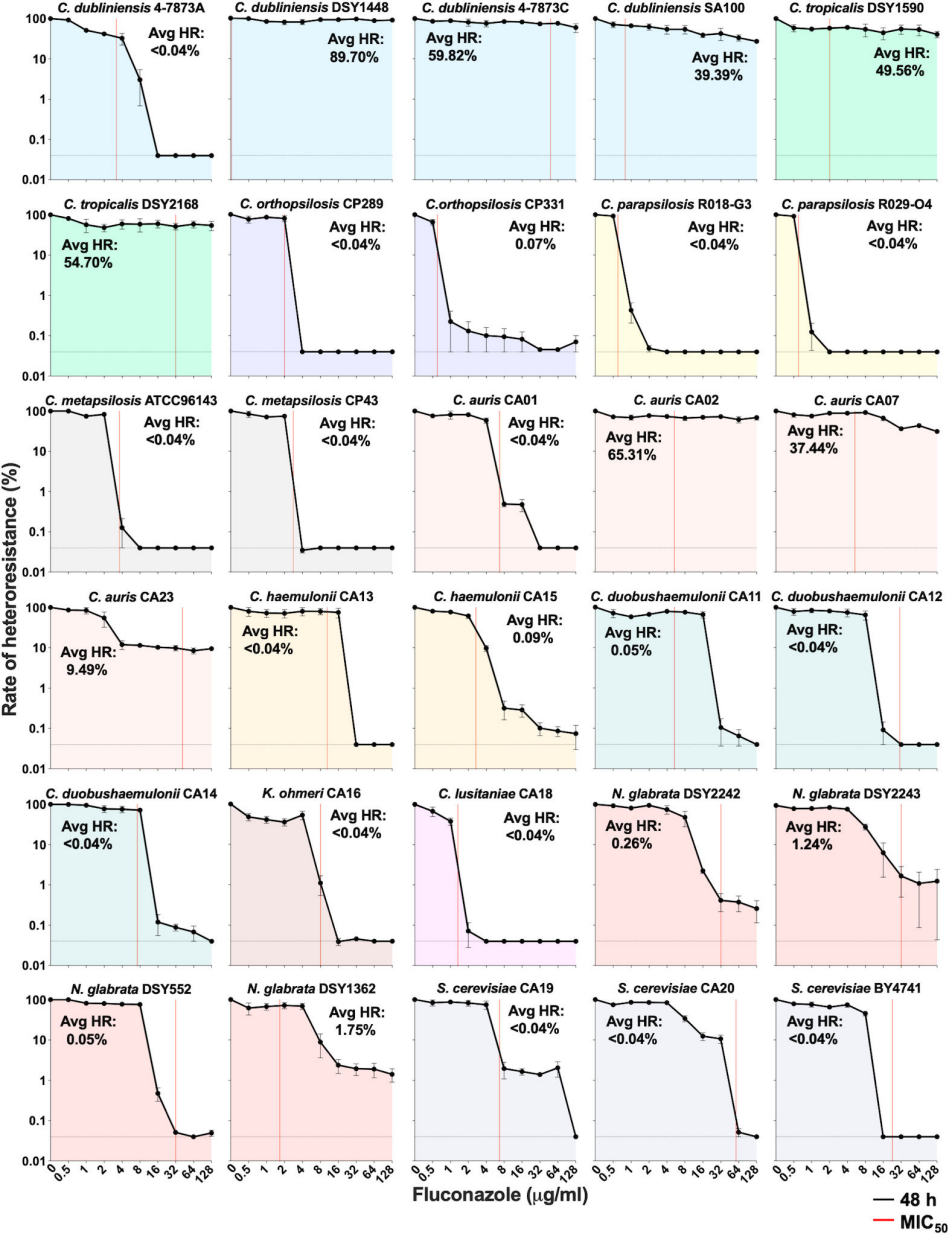
PAP profiles of a collection of 30 isolates from other yeast species. Assays were performed as described in Fig. 2. Black lines show average heteroresistance rates determined after 48 h incubation. Heteroresistance rates were calculated by taking the average growth on PAP at 48 h for FLC concentrations equal to or greater than 10-fold the MIC_50_ of the parent isolate (Avg HR). When such concentrations were outside of the dynamic range of the assay, the rate observed at 128 µg/mL FLC was used instead. The dotted lines indicate the detection limit of the assay (0.04%), and the red lines show MIC_50_ levels. Error bars show the standard error of the mean (S.E.M.) from three biological replicates. Graphs are color-coded according to the species.

To assess the use of the PAP assay in other yeast species, we gathered a collection of 30 isolates belonging to *Candida dubliniensis*, *Candida tropicalis*, *Candida orthopsilosis*, *C. parapsilosis*, *Candida metapsilosis*, *C. auris*, *Candida haemuloniii*, *Candida duobushaemulonii*, *Kodamaea ohmeri*, *Candida lusitaniae*, *N. glabrata*, and *Saccharomyces cerevisiae* species (Table 1). The strains selected displayed susceptibility levels lower than 64 µg/mL FLC, with MIC_50_ levels ranging from 0.25 to 57.6 µg/mL FLC (Fig. S5A), which allowed the detection of potential heteroresistant colonies on PAP assays at a minimum of two concentrations (64 and 128 µg/mL FLC). These isolates showed a wide range of FLC tolerance levels when tested in MIC assays, ranging from 0.13 to 0.86 SMG (Fig. S5B). Testing this collection using the PAP method revealed diverse growth profiles, with potential heteroresistant subpopulations encompassing 0.05%–89.7% of the population (Fig. 5). Isolates showed diverse AUC values, both well below and well above those of the *C. albicans* reference strain SC5314, which generally reflected heteroresistant rates measured in PAP assays (Fig. S5C). While no significant correlation was observed between the average heteroresistance rate of these isolates and susceptibility (MIC_50_, Spearman correlation test, *r* = 0.0126, *P* = 0.947; Fig. S6A), a modest positive correlation was observed with tolerance (SMG, Spearman correlation test, *r* = 0.562, *P* = 0.001; Fig. S6B), indicating that these phenomena could be linked. Interestingly, only isolates with high tolerance levels displayed some degree of heteroresistance.

We found that seven isolates from *C. dubliniensis* (DSY1448, 4-7873C, and SA100), *C. tropicalis* (DSY1590 and DSY2168), and *C. auris* (CA02 and CA07) displayed high heteroresistance rates, ranging from 37.44% to 89.7% of the population (Fig. 5). Susceptibility testing of colonies growing on PAP assays at 128 µg/mL FLC revealed that ~51% (57/112) of the colonies tested had higher resistance (≥1.25× MIC_50_) relative to the corresponding parent strains (Fig. S7A). For this set of isolates, most colonies (64/78, ~82%) for which tolerance could be measured showed similar tolerance to that of their corresponding parent isolate, with only ~18% of the colonies displaying increased tolerance (≥1.25× SMG; Fig. S7B). Seven isolates from *C. orthopsilosis* (CP331), *C. auris* (CA23), *C. haemulonii* (CA15), *C. duobushaemulonii* (CA11), and *N. glabrata* (DSY2242, DSY2243, DSY552, and DSY1362) showed low levels of heteroresistance, ranging from 0.05% to 9.49% of the total population (Fig. 5). For these isolates, 78.8% (67/85) of the colonies recovered from PAP assays were more resistant than the parent isolate (≥1.25× MIC_50_; Fig. S7A). Two isolates from this set (CP331 and DSY1362) formed colonies with increased tolerance (≥1.25× SMG), while most colonies (26/41, 63.4%) showed tolerance levels similar to those of the parent isolate (Fig. S7B). Finally, for the remaining 16 isolates, including those from *C. dubliniensis* (4-7873A), *C. orthopsilosis* (CP289 and CP331), *C. parapsilosis* (R018-G3 and R029-O4), *C. metapsilosis* (ATCC96143 and CP43), *C. auris* (CA01), *C. haemulonii* (CA13), *C. duobushaemulonii* (CA12 and CA14), *K. ohmeri* (CA16), *C. lusitaniae* (CA18), and *S. cerevisiae* (CA19, CA20, and BY4741), no heteroresistant colonies could be recovered (<0.04% of the population; Fig. 5), indicating either the limitation of our current testing method or the absence of heteroresistant cell formation in these isolates.

Thus, PAP assays were able to detect the presence of heteroresistance in 14 out of the 30 isolates examined. Most of the colonies recovered (124/197, 62.9%) from these assays showed higher resistance relative to the parent isolates, while only a small fraction displayed increased tolerance (26/119, 21.85%), therefore validating this approach. The frequency of recovery of resistant colonies was dependent on the genetic background of the isolate rather than on the species of origin. Overall, these results indicate that azole heteroresistance is prevalent among other *Candida* species and that the PAP method coupled with additional validation of colonies can be used to detect and measure this phenomenon.

## DISCUSSION

Heteroresistance can compromise antimicrobial treatment and complicate the selection of optimal therapeutic strategies resulting in clinical failure and relapse. In bacterial populations, this leads to the use of combination therapies or higher doses of antimicrobials to ensure effective treatment against both susceptible and resistant subpopulations (30, 31, 34, 46). In the long term, heteroresistance can contribute to the development and spread of drug resistance. In fungal pathogens, heteroresistance is emerging as a significant challenge in the context of antifungal treatment (20–23, 25, 27, 35), requiring precise and efficient methods for identification and measurement. Here, we present both optimized and novel approaches for detecting and quantifying azole heteroresistance in *Candida* species, highlighting the advantages and limitations of each method.

Disk diffusion assays are routinely employed to measure susceptibility and tolerance (36). Here, we show that they can also be used to identify the presence of heteroresistant colonies (Fig. 1). However, this method falls short in assessing the frequency of heteroresistance, a critical parameter in understanding the dynamics of resistance development.

To address these limitations, we argue for the use of PAP and single-cell assays, which offer a comprehensive assessment of rates of heteroresistance among susceptible populations. We note that single-cell assays involve a lengthier protocol with a higher detection limit relative to PAP assays (0.13%–0.26% versus 0.04% of the population, respectively) but can reveal a wide spectrum of adaptation to antifungal exposure, as illustrated by the diversity of susceptibility and tolerance levels of the isolates recovered (Fig. S3). Furthermore, we adapted the conventional PAP to a short PAP assay, a simplified and faster method for measuring heteroresistance (Fig. 3). The short PAP assay significantly reduces labor demands, making it amenable for the screening of large numbers of isolates. Importantly, we reveal moderate but significant correlations in the heteroresistance rates determined with these different methods (Fig. S2C and D).

Our findings also highlight that environmental conditions are crucial for the accurate assessment of this phenomenon and emphasize the importance of species-specific considerations. Indeed, there are substantial differences in how heteroresistance is measured in different fungal species (20, 23, 25). For example, FLC heteroresistance in *N. glabrata* was measured on YAG agar after 24 h of growth following incubation of PAP plates at 30°C for 24 h (20). In our study, PAP assays identified heteroresistant subpopulations in this species following incubation on YPD plates at 30°C for 48 h, while no visible colonies were detectable at 24 h. A *C. neoformans* study used FLC YPD plates, which were incubated at 30°C for 5 days (23). In contrast, a *C. parapsilosis/C. auris* study used echinocandin-supplemented YPD plates, which were incubated for 2–3 days at 35°C (25). The variations in medium, incubation time, and temperature emphasize the need for species-specific and antifungal-specific tailored methodologies. Differences in the intrinsic growth rates of these fungal species under different conditions further highlight the complexity of measuring these drug responses. For example, in *N. glabrata*, the presence of heteroresistant cells was apparent on YAG medium at 24 h (20), whereas these subpopulations required 48 h for detection on YPD (Fig. 5). This could be due to intrinsic differences between isolates, an increased propensity for developing azole resistance, and/or a faster emergence of heteroresistance, potentially allowing for earlier detection.

The high diversity in heteroresistance rates observed here with different *C. albicans* isolates is likely due to both the ability of cells to grow in the presence of high drug concentrations as well as the stability of heteroresistant cells once they arise. This was most apparent in single-cell assays, where both the frequency of wells with proficient growth as well as the rate of recovery of heteroresistant cells varied substantially between isolates (Fig. 4B and C). It remains unknown what fraction of heteroresistant cells reverted to high susceptibility during validation by MIC assays. An additional variable is the presence of tolerant cells, which can confound the number of colonies and wells recovered, respectively. Indeed, a subset of tolerant isolates was detected among *C. albicans* isolates recovered from single-cell assays (Fig. S3B). Therefore, subsequent validation of recovered isolates in susceptibility assays is essential for establishing the size of the heteroresistant subpopulation. More precise methods could be developed to measure this phenomenon, including through the identification of genetic markers that can predict the ability of isolates to form heteroresistant cells. We were also surprised by the extensive diversity in MIC_50_ levels of isolates recovered from single-cell assays after a short exposure to high FLC concentrations (Fig. S3A), indicating that this assay could reveal a higher diversity of adaptation strategies than previously reported assays in which whole populations are exposed to similar doses of azoles (47, 48).

Analysis of isolates from other yeasts revealed that many other *Candida* species also display azole heteroresistance and that PAP assays can detect the presence of heteroresistant subpopulations (Fig. 5). However, additional validation of colonies growing on PAP assays is recommended for validating the increased resistance of these subpopulations. While most of the colonies growing on PAP plates were indeed more resistant, a subset of them showed increased tolerance (Fig. S7B). We cannot exclude the possibility that isolates could have lost resistance once removed from the selective medium, as it is often the case with heteroresistant isolates (49). Across the collection, heteroresistance rates did not appear associated with phylogeny, although the limited number of isolates examined does not allow for the direct testing of any correlations. Second, additional optimization might be required for each species to improve the rate of recovery by potentially adjusting the growth medum, incubation time, or temperature.

Our data further emphasize the importance of the experimental conditions used to monitor different drug responses as experiments performed on solid RPMI medium failed to detect both tolerance and heteroresistance in *C. albicans* (Fig. S4). Indeed, medium composition, incubation time, temperature, pH, shaking, and inoculum size have been previously shown to impact drug susceptibility (50, 51). Some of these parameters have been specifically linked to variations in tolerance. Trailing growth (or tolerance) appears to vary with medium, temperature, pH, glucose concentration, type of microtiter plates, and shaking speed (28, 48, 52–56). To some extent, the differences seen here between YPD (pH ~ 5) and RPMI (pH 7) could be attributed to differences in pH. Marr and colleagues (56) showed that a pH below neutrality decreases tolerance in both YPD and RPMI. In line with this report, we previously found that FoG and SMG levels were reduced in YPD at pH 4.5 relative to pH 7, while high temperatures (39°C) also reduced tolerance (28). Therefore, the absence of shaking, the higher temperature, the different medium composition, and the increased pH are all likely to impact our drug response measurements and could account for the absence of heteroresistant colonies on RPMI versus YPD agar (Fig. S4).

It is also worth noting that the diversity in heteroresistant rates is likely a function of the genetic background of the respective isolate impacting both the rate of emergence of heteroresistant cells as well as their stability in the absence of selective pressures. The fitness costs associated with increased drug resistance could vary depending on the type of genetic and/or epigenetic changes underlying heteroresistance. Indeed, in bacterial populations, heteroresistant cells emerge through the acquisition of genetic mutations as well as via gene amplifications, the latter of which being intrinsically unstable (49). The high rates of heteroresistance observed here are not consistent with mutational events occurring during such short time intervals of *in vitro* growth. For *C. albicans*, the average mutation rate was calculated to ~1.2 × 10^−10^ base substitutions per base pair per generation (57), although mutation rates have not been formally measured under high FLC exposure. However, other genetic events previously observed during azole stress could contribute to heteroresistance. For example, the formation of tetraploid intermediates that progress into mitotic collapse and unequal DNA segregation leading to chromosomal aneuploidy could represent one scenario (58).

In conclusion, our study introduces both new and refined methodologies for identifying and measuring heteroresistance in *C. albicans* susceptible populations subjected to azole stress. The comparative analysis of different assays provides a nuanced understanding of their advantages and limitations. Recognizing the environment- and species-specific variations in growth dynamics and response to antifungal agents is crucial for characterizing heteroresistance and for developing targeted strategies to combat it in diverse *Candida* species. This work lays the foundation for future investigations aiming to unravel the intricate mechanisms underlying heteroresistance and potentially optimize antifungal interventions.

## SUPPLEMENTAL MATERIAL

Figure S1 spectrum.04041-23-s0001.tiff Drug response measurements for the set of 12 *C. albicans* isolates.

Figure S2 spectrum.04041-23-s0002.tiff Heteroresistance rate correlations for *C. albicans* isolates.

Figure S3 spectrum.04041-23-s0003.tiff FLC susceptibility and tolerance for isolates recovered from Single Cell Assays.

Figure S4 spectrum.04041-23-s0004.tiff RPMI medium cannot accurately distinguish between susceptible, tolerant, and heteroresistant isolates.

Figure S5 spectrum.04041-23-s0005.tiff Drug response measurements of isolates from other species.

Figure S6 spectrum.04041-23-s0006.tiff Heteroresistance rate correlations for isolates from other species.

Figure S7 spectrum.04041-23-s0007.tiff Validation of colonies recovered from PAP assays of isolates from other species.

ASM does not own the copyrights to Supplemental Material that may be linked to, or accessed through, an article. The authors have granted ASM a non-exclusive, world-wide license to publish the Supplemental Material files. Please contact the corresponding author directly for reuse.
