# Supplementary figures and images for: Approaches for identifying and measuring heteroresistance in azole-susceptible Candida isolates
Source: Microbiol Spectr. 2024 Mar 14;12(4):e04041-23. doi: 10.1128/spectrum.04041-23 (PMC10986555; doi:10.1128/spectrum.04041-23)

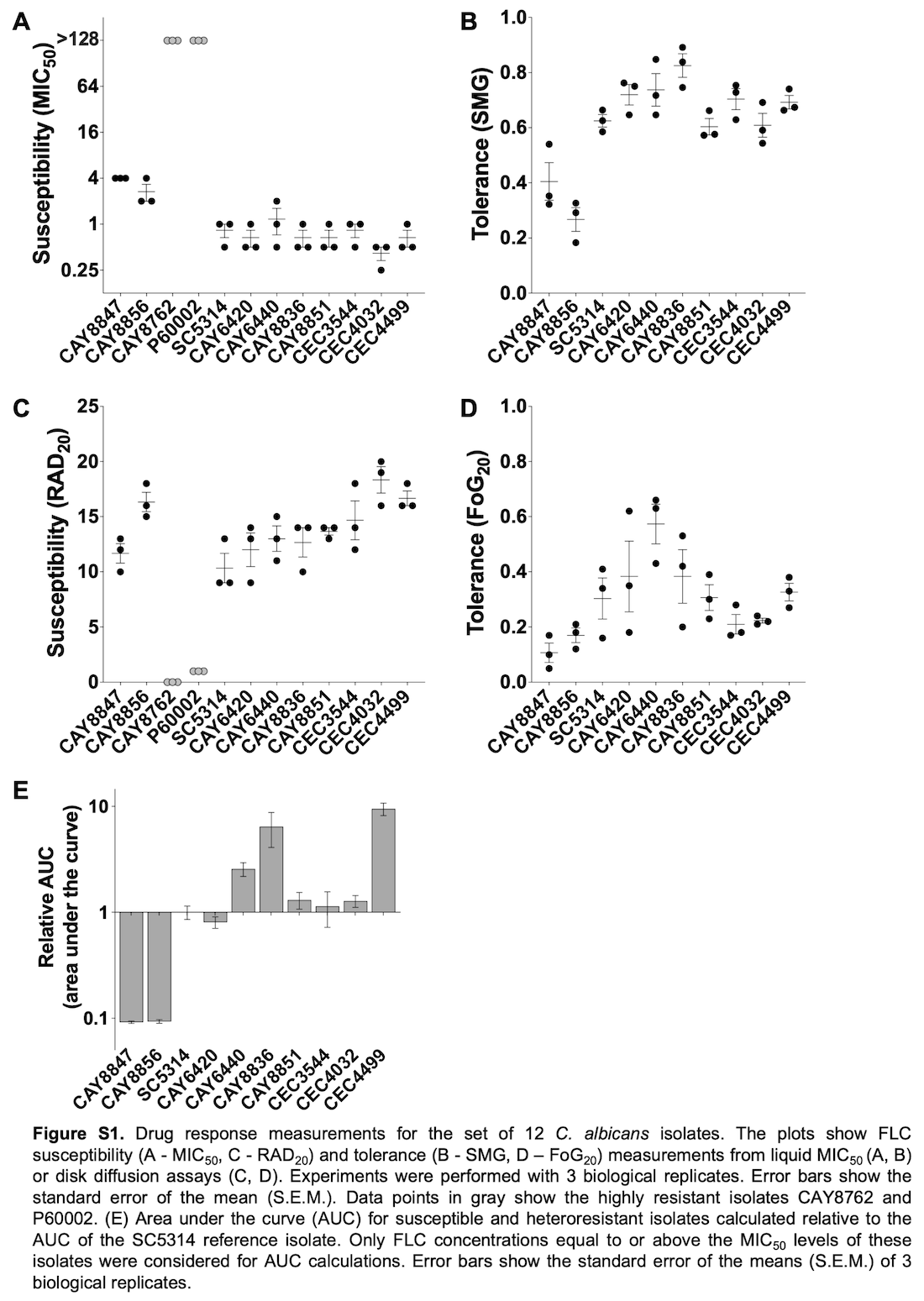

Supplement: Figure S1 — Drug response measurements for the set of 12 C. albicans isolates. [file spectrum.04041-23-s0001.tiff]

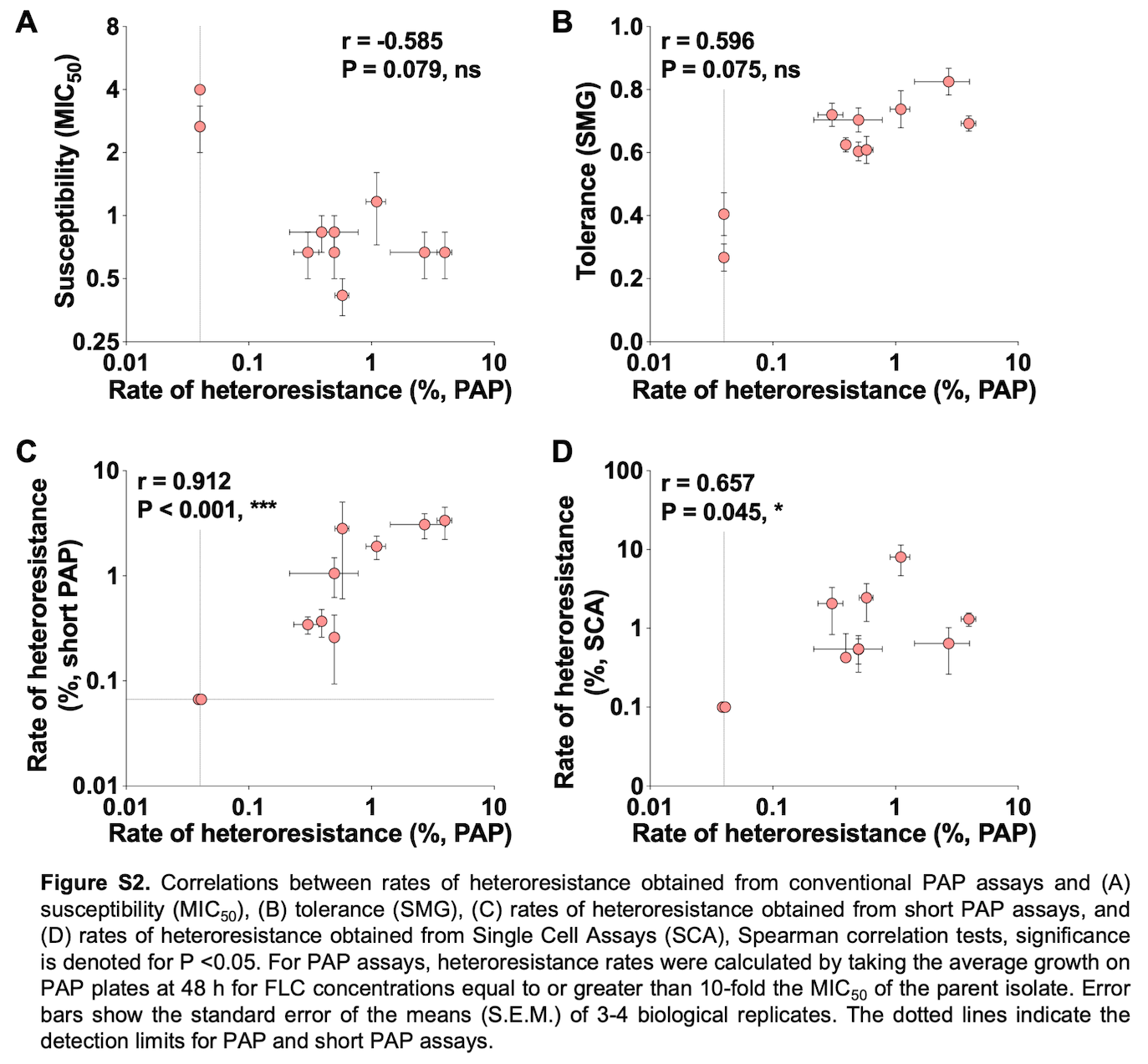

Supplement: Figure S2 — Heteroresistance rate correlations for C. albicans isolates. [file spectrum.04041-23-s0002.tiff]

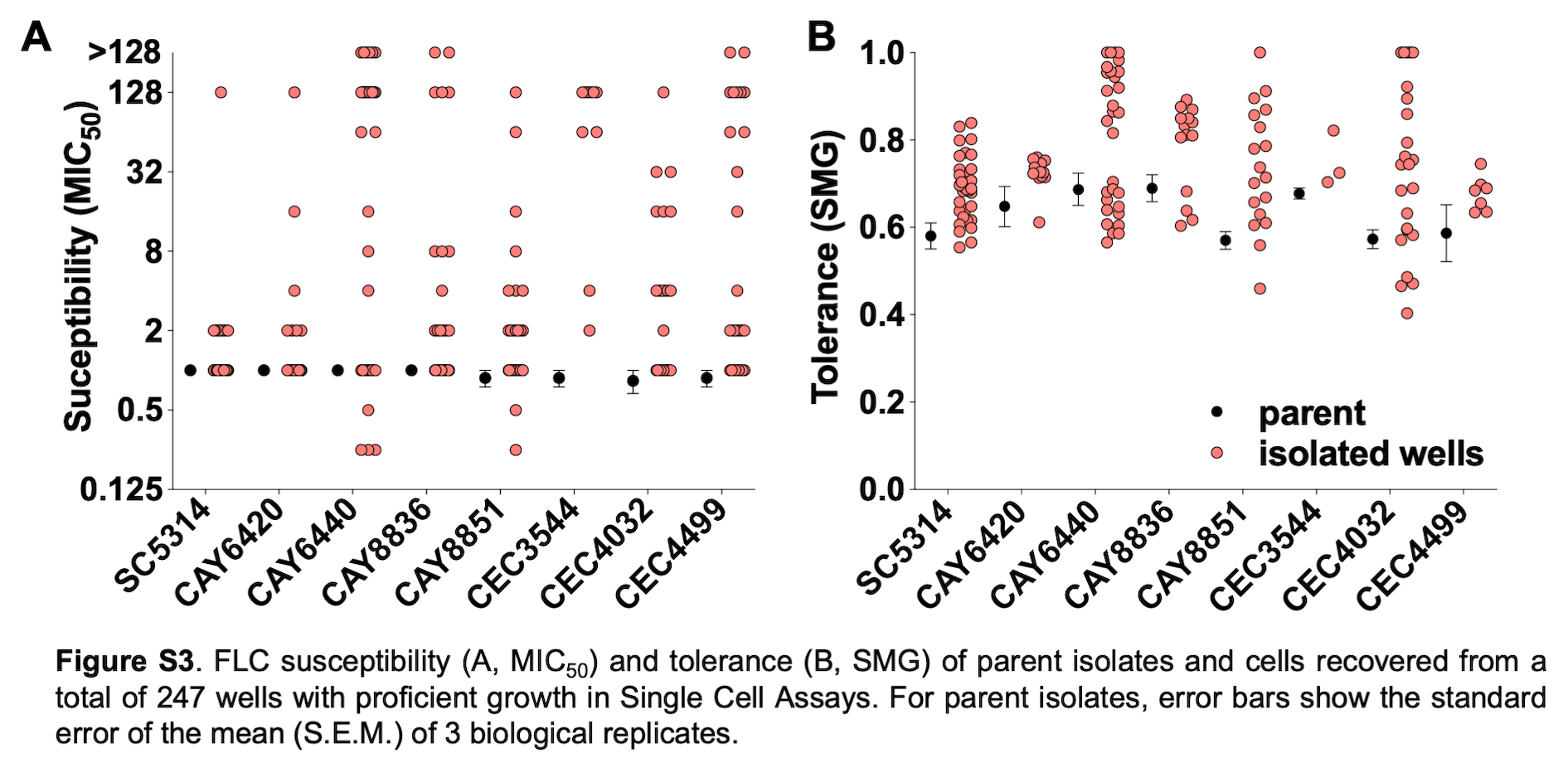

Supplement: Figure S3 — FLC susceptibility and tolerance for isolates recovered from Single Cell Assays. [file spectrum.04041-23-s0003.tiff]

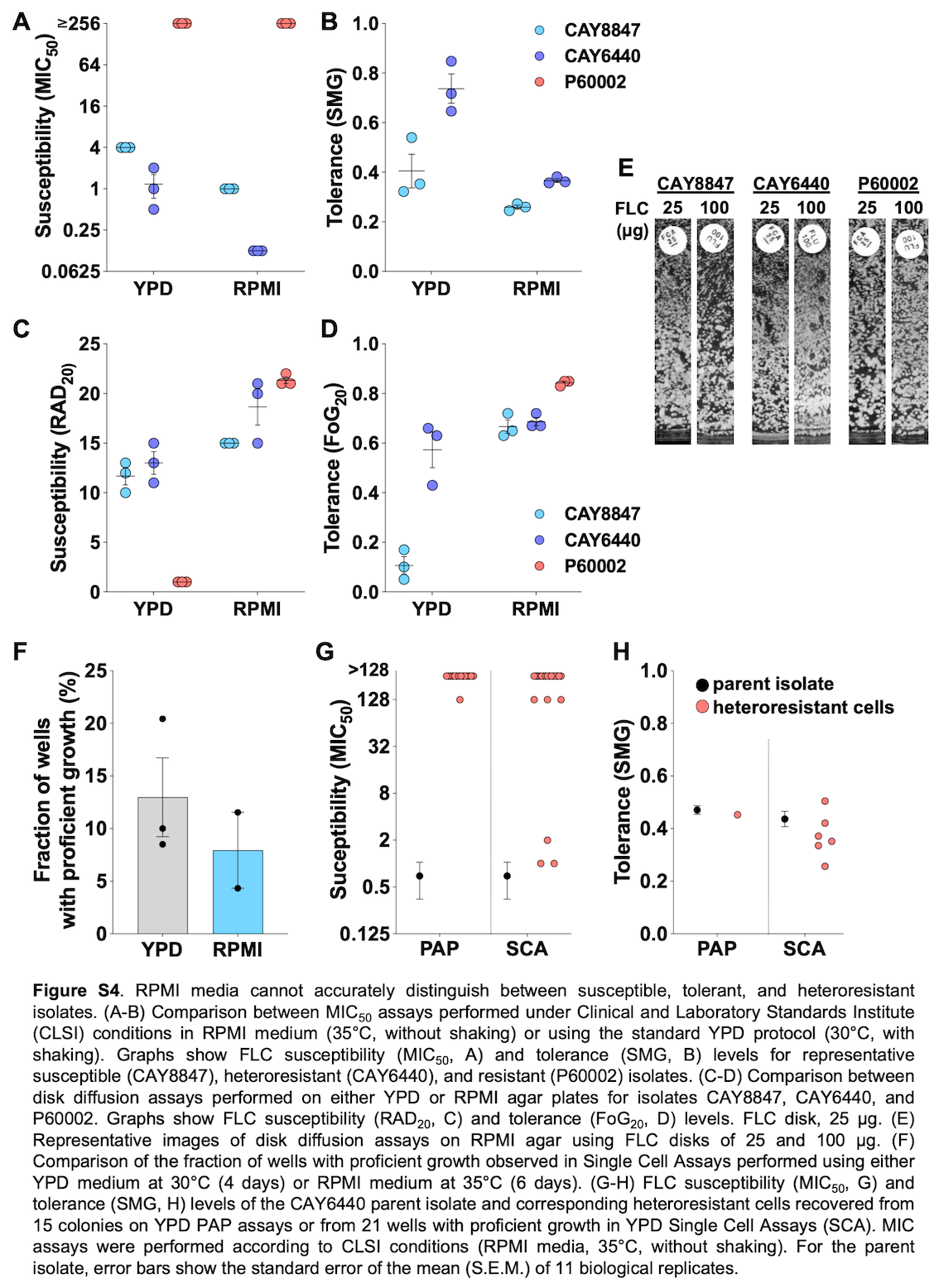

Supplement: Figure S4 — RPMI medium cannot accurately distinguish between susceptible, tolerant, and heteroresistant isolates. [file spectrum.04041-23-s0004.tiff]

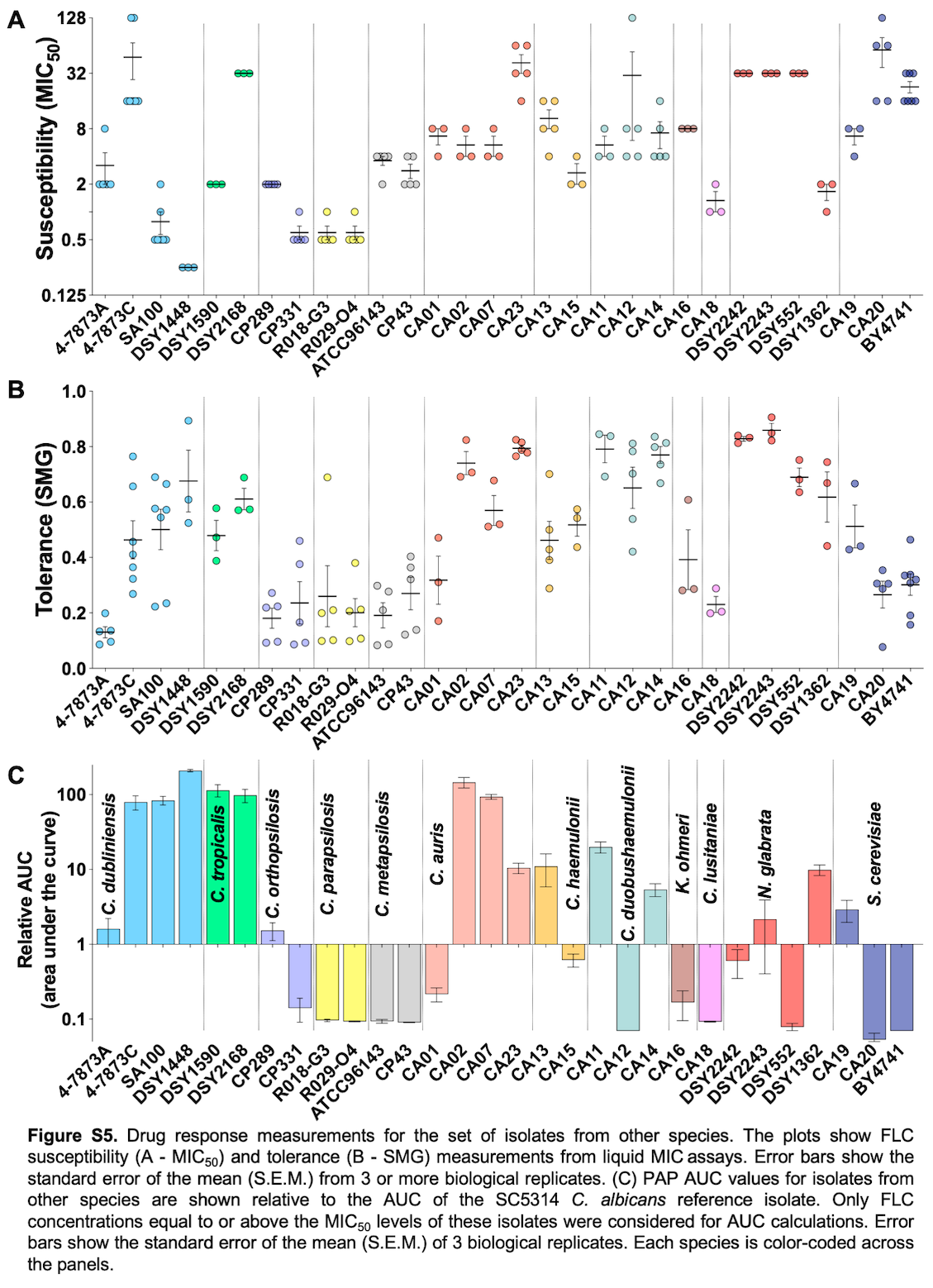

Supplement: Figure S5 — Drug response measurements of isolates from other species. [file spectrum.04041-23-s0005.tiff]

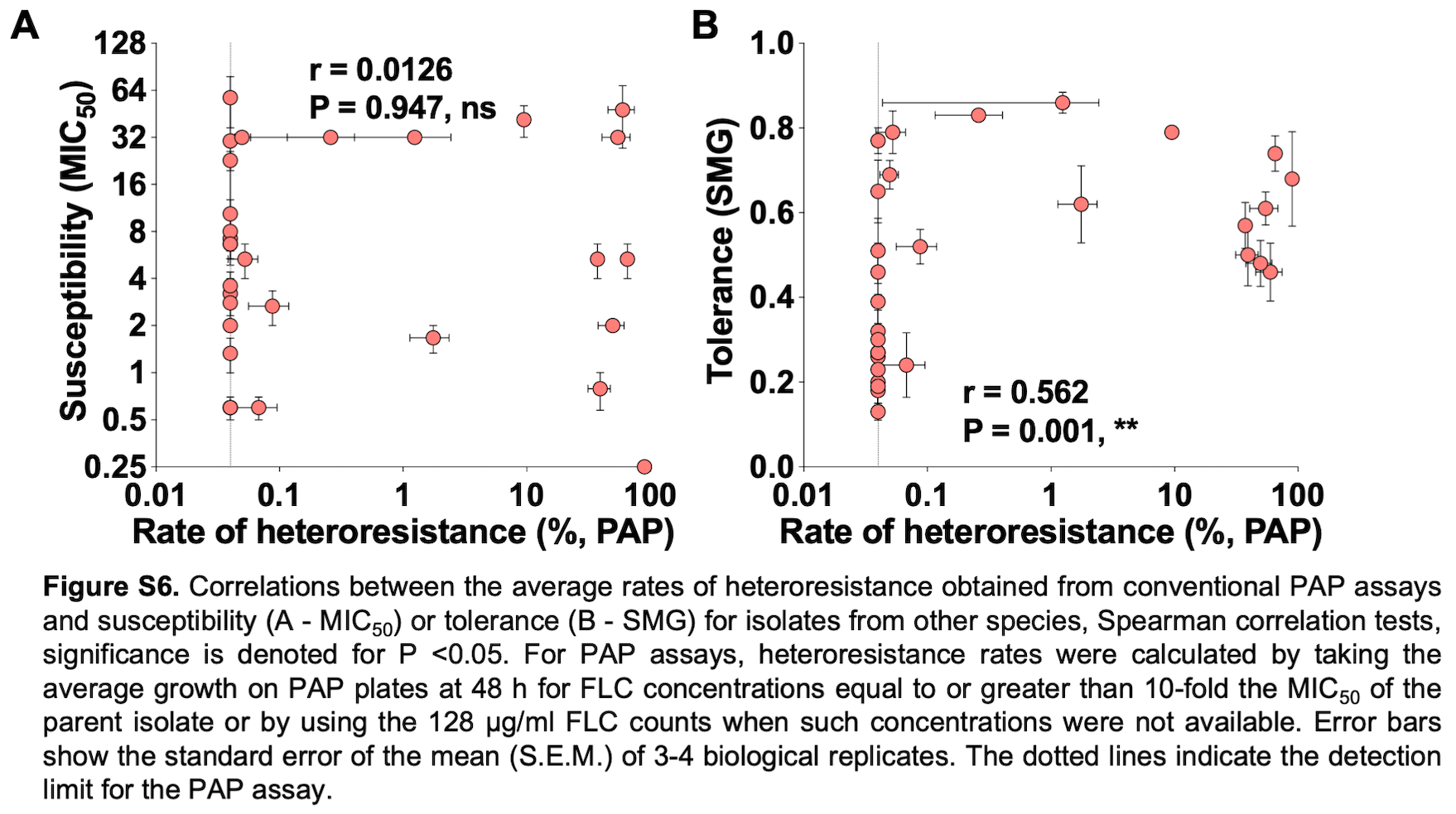

Supplement: Figure S6 — Heteroresistance rate correlations for isolates from other species. [file spectrum.04041-23-s0006.tiff]

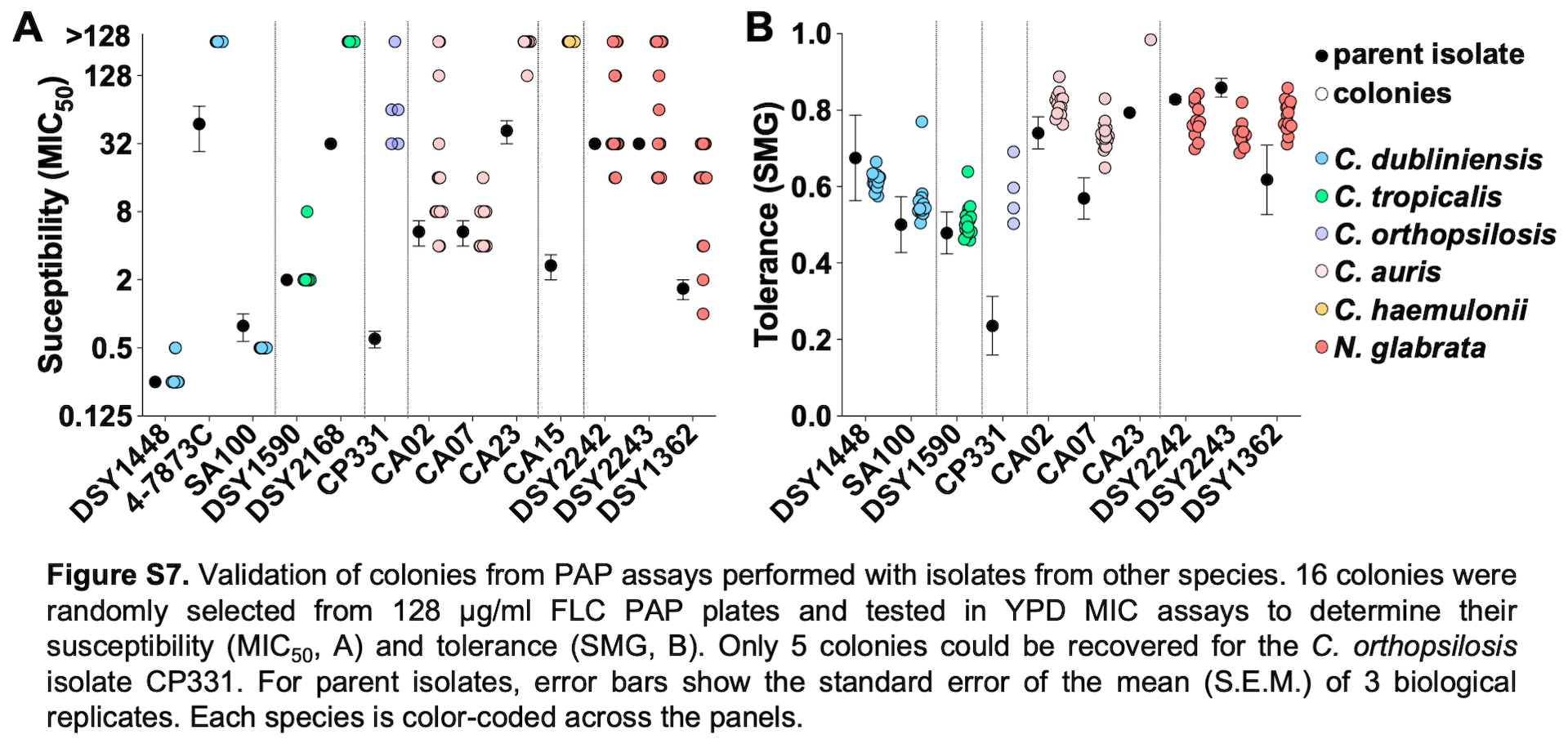

Supplement: Figure S7 — Validation of colonies recovered from PAP assays of isolates from other species. [file spectrum.04041-23-s0007.tiff]
